# A comprehensive multi-omics analysis reveals unique signatures to predict Alzheimer’s disease

**DOI:** 10.3389/fbinf.2024.1390607

**Published:** 2024-06-19

**Authors:** Michael Vacher, Rodrigo Canovas, Simon M. Laws, James D. Doecke

**Affiliations:** ^1^ The Australian eHealth Research Centre, CSIRO Health and Biosecurity, Kensington, WA, Australia; ^2^ Centre for Precision Health, Edith Cowan University, Joondalup, WA, Australia; ^3^ The Australian eHealth Research Centre, CSIRO Health and Biosecurity, Parkville, VIC, Australia; ^4^ Collaborative Genomics and Translation Group, School of Medical and Health Sciences, Edith Cowan University, Joondalup, WA, Australia; ^5^ Curtin Medical School, Curtin University, Bentley, WA, Australia; ^6^ The Australian eHealth Research Centre, CSIRO Health and Biosecurity, Herston, QLD, Australia

**Keywords:** Alzheimer disease, systems biology, multi omics analysis, biomarkers prediction, bioinformatics

## Abstract

**Background:**

Complex disorders, such as Alzheimer’s disease (AD), result from the combined influence of multiple biological and environmental factors. The integration of high-throughput data from multiple omics platforms can provide system overviews, improving our understanding of complex biological processes underlying human disease. In this study, integrated data from four omics platforms were used to characterise biological signatures of AD.

**Method:**

The study cohort consists of 455 participants (Control:148, Cases:307) from the Religious Orders Study and Memory and Aging Project (ROSMAP). Genotype (SNP), methylation (CpG), RNA and proteomics data were collected, quality-controlled and pre-processed (SNP = 130; CpG = 83; RNA = 91; Proteomics = 119). Using a diagnosis of Mild Cognitive Impairment (MCI)/AD combined as the target phenotype, we first used Partial Least Squares Regression as an unsupervised classification framework to assess the prediction capabilities for each omics dataset individually. We then used a variation of the sparse generalized canonical correlation analysis (sGCCA) to assess predictions of the combined datasets and identify multi-omics signatures characterising each group of participants.

**Results:**

Analysing datasets individually we found methylation data provided the best predictions with an accuracy of 0.63 (95%CI = [0.54–0.71]), followed by RNA, 0.61 (95%CI = [0.52–0.69]), SNP, 0.59 (95%CI = [0.51–0.68]) and proteomics, 0.58 (95%CI = [0.51–0.67]). After integration of the four datasets, predictions were dramatically improved with a resulting accuracy of 0.95 (95% CI = [0.89–0.98]).

**Conclusion:**

The integration of data from multiple platforms is a powerful approach to explore biological systems and better characterise the biological signatures of AD. The results suggest that integrative methods can identify biomarker panels with improved predictive performance compared to individual platforms alone. Further validation in independent cohorts is required to validate and refine the results presented in this study.

## Introduction

Alzheimer’s disease (AD) is a complex neurodegenerative disorder, clinically characterized by progressive cognitive decline, memory loss, and impairment in daily functioning. It is the most common cause of dementia worldwide, affecting millions of individuals and posing a significant burden on healthcare systems and society (Brookmeyer et al., 2007; Nichols et al., 2022). The aetiology of AD is multifactorial, involving a combination of genetic, environmental, and epigenetic factors (Breijyeh and Karaman, 2020). Currently, AD diagnosis involves a combination of medical history, physical examinations, neuropsychological tests, and cerebrospinal fluid analysis in some cases. Imaging serves as a supportive tool and helps rule out other causes of cognitive impairment. However, a comprehensive evaluation by a professional is essential for an accurate diagnosis (Rodrigue, 2013; DeTure and Dickson, 2019; Porsteinsson et al., 2021). Given these diagnostic challenges, understanding the underlying biological processes and identifying reliable biomarkers for early detection and accurate diagnosis are crucial for developing effective therapeutic strategies and interventions.

In recent years, the continuous advancements in high-throughput technologies have provided unprecedented opportunities to explore complex disorders at the molecular level. These technological improvements have not only increased the diversity of omics platforms available but also their resolution. While the analysis of single omics platform provides a unique perspective, capturing specific molecular changes associated with a trait of interest, this approach also limits our understanding of the complete molecular landscape underlying complex pathogenesis.

To address this limitation, there has been a growing interest in the integration of data across multiple omics platforms (i.e., “multi-omics”), to comprehensively explore the interactions and alterations occurring at multiple biological levels. Multi-omics integrations aim to capture a broader view of biological systems and therefore holds great promise in unravelling the complex molecular interplay across biological domains (Ivanisevic and Sewduth, 2023). This knowledge is essential to enhance our understanding of the underlying mechanisms driving complex disorders such as AD and facilitate the development of personalised and targeted therapies.

In this study, we present an integrated analysis of four omics platforms, including single nucleotide polymorphism (SNP), methylation (CpG), transcriptomic (RNA), and proteomics data, to characterise the biological signatures of AD. Leveraging a well-characterised cohort from the Religious Orders Study and Memory and Aging Project (ROSMAP) (Bennett et al., 2012Bennett et al., 2012), consisting of individuals categorized as no cognitive impairment (NCI), mild cognitive impairment (MCI), and AD patients, we employed integrative approaches to predict the disease status based on each omics dataset individually. Subsequently, we utilized a variation of the generalized canonical correlation analysis (sGCCA) (Kettenring, 1971; Tenenhaus et al., 2014)to integrate the four datasets and identify multi-omics signatures specifically associated with AD participants.

## Materials and methods

### Participants and clinical characterisation

Data used in the preparation of this article were obtained from the Religious Orders Study and Memory and Aging Project (ROSMAP) (Bennett et al., 2012). The synapse portal (https://adknowledgeportal.synapse.org/) offers comprehensive list of data, we used four different datasets from this resource, including: proteomics (https://doi.org/10.7303/syn10468856), epigenetics (DNA methylation array, https://doi.org/10.7303/syn3157275), genomic variants (SNP Array, https://doi.org/10.7303/syn3157325) and gene expression (RNAseq from bulk brain, https://doi.org/10.7303/syn3388564). These four datasets were selected as they provided the largest number of overlapping samples (N = 455). Participants were divided into two groups, based on their clinical characterisation at death, generating a case/control binary outcome. Specifically, participants were considered “*cases*” when the most likely clinical diagnosis at the time of death was AD or MCI (Mild Cognitive Impairment) and “*control*” when diagnosed as NCI (No Cognitive Impairment). The participants’ data include phenotypic information relevant to AD, such as the Braak stage, which classifies AD progression based on neurofibrillary tangle pathology throughout the brain (Braak and Braak, 1991; Braak et al., 2006) and the Consortium to Establish a Registry for Alzheimer’s Disease (CERAD) score, a standardized method for assessing the severity of neuritic plaques (Fillenbaum et al., 2008). Detailed demographics are summarised in Table 1.

**TABLE 1:** Population demographics.

	Control (N = 148)	Case (N = 307)	*p*-value
Sex			
Female	85 (57.4%)	202 (65.8%)	0.0971
Male	63 (42.6%)	105 (34.2%)	
Age (years)			
Mean (SD)	82.9 (4.79)	85.4 (4.28)	<0.001
Median [Min, Max]	84.2 [67.4, 89.7]	86.7 [70.3, 90.0]	
Education (years)			
Mean (SD)	16.4 (3.39)	16.4 (3.47)	0.973
Median [Min, Max]	16.0 [10.0, 25.0]	16.0 [5.00, 28.0]	
*APOE* ε4			
Absent	126 (85.1%)	217 (70.7%)	<0.001
Present	22 (14.9%)	90 (29.3%)	
Braak stage			
I	6 (4.1%)	1 (0.3%)	<0.001
II	25 (16.9%)	11 (3.6%)	
III	17 (11.5%)	24 (7.8%)	
IV	54 (36.5%)	85 (27.7%)	
V	41 (27.7%)	97 (31.6%)	
VI	5 (3.4%)	83 (27.0%)	
CERAD			
positive	60 (40.5%)	220 (71.7%)	<0.001
negative	88 (59.5%)	87 (28.3%)	

*p* values determined by *t*-test for continuous variable or Chi square for categorical variables. N number, HC, healthy control; MCI, mild cognitive impairment; AD, Alzheimer’s disease, *APOE* ε4 apolipoprotein ε4 allele, CERAD, Consortium to Establish a Registry for Alzheimer’s Disease.

### Data preparation and feature reduction

The analyses were restricted to samples present in all four datasets investigated. Each dataset was therefore limited to these samples and was further prepared as follows.

#### RNAseq

Samples were extracted using Qiagen’s miRNeasy mini kit and the RNase free DNase Set. They were quantified by Nanodrop and quality was evaluated by Agilent Bioanalyzer. The initial dataset consisted of 642 samples and 55,889 transcripts, stored as raw FPKM (Fragments Per Kilobase of transcript per Million mapped reads) values. After removing non-overlapping samples, we discarded lowly expressed transcripts based on the threshold of geometric mean of (FPKM + 0.1) < 1. FPKM values were then transformed to log2 scale. To further reduce the number of features, we built an elastic net regression model using the case/control phenotype as target variable. The initial data was separated into two subsets (training set = 70% [N = 318], test set = 30% [N = 137]) and the model’s training was performed using 10-fold cross-validation and averaged the obtained classification error rate across 50 repetitions to identify the optimal parameters (lambda). The trained model was then used to identify and remove transcripts not contributing to the phenotype’s prediction (zero coefficient). The final data consisted of 455 samples and 91 transcripts.

#### Proteomics

Proteomics assay was performed using frozen tissue from dorsolateral prefrontal cortex (DLPFC) on a nano ACQUITY UPLC coupled to TSQ Vantage MS instrument. Samples were prepared using standard protocol described in the original publications (Petyuk et al., 2010; Andreev et al., 2012). The initial dataset contained 1,191 samples and 121 proteins. Control probes and samples were removed, resulting in a final set consisting of 455 samples and 119 proteins.

#### SNP

Two batches of genotype data are available in ROS and MAP studies. The first batch was generated using the Affymetrix GeneChip 6.0 (Affymetrix, Inc., Santa Clara, CA, United States) and contained 1,709 individuals. The second batch used the Illumina HumanOmniExpress (Illumina, Inc., San Diego, CA, United States) on 382 samples. Both batches underwent the same quality control (QC) analysis, as described in (De Jager et al., 2012). After non-overlapping samples were removed the two sets were merged and the quality controlled. The QC assessment included exclusion of samples with genotype success rate <95%, discordance between inferred and reported gender, and excess inter/intra heterozygosity. SNP-level quality control assessment included exclusion of SNPs with Hardy-Weighberg equilibrium (*p* < 0.001), MAF <0.01, genotype call rate <0.95, misshap test < 1 × 10^−9^. Population outliers were identified and removed using Eigenstrat (Price et al., 2006) with default parameters.

To further reduce the number of SNP, we employed logistic regression models using the case/control status as a binary outcome. Models’ covariates included education (years), the presence/absence of the *APOE* ε4 allele (binary) and the first 3 principal components of a principal component analysis (PCA), to control for potential population structure. Results from the logistic regressions were adjusted for multiple testing using the Benjamin-Hochberg method. SNP with *p*-values below 0.05 were considered significant and selected for the downstream analyses. The final data included 455 samples and 145 SNPs.

#### Methylation

The initial data contained 741 samples (prefrontal cortex) and 420,132 cpgs, collected using the Illumina HumanMethylation450 BeadChip. Data generation method was described in (De Jager et al., 2014). To reduce the number of features prior to integration, the same method as for the RNAseq data was used. The dataset was split into two subsets (training set = 70% [N = 318], test set = 30% [N = 137]) and used to train an elastic net regression model. Training phase used 10-fold cross-validation and averaged the obtained classification error rate across 50 repetitions to identify the optimal parameters (lambda). The trained model was used to identify and remove probes not contributing to the phenotype’s prediction (zero coefficient). The final data consisted of 455 samples and 91 CpGs.

### General analytical pipeline

To facilitate comparisons, the same analytical pipeline was used to assess the predictive capabilities of each individual omics dataset and the integrated dataset. First, participants were randomly divided in two groups (training set = 70% [N = 318], test set = 30% [N = 137]). An initial model was then built and tuned using the training data only. Two different models were used depending on the type of the dataset (single omics or integrated), as detailed in the following section. In the context of this study, the tuning phases allowed the identification of the optimal number of components as well as the optimal number of features to select in each of these components. These parameters were considered optimal when they provided the smallest Balanced Error Rate (BER). Tuning phases were performed using a 10-fold, 50 repeats procedure, to limit the impact of the randomly allocated folds at each repetition. The models were then trained on the training data only. Finally, the trained models were used to perform predictions on the *test* set (unseen data) and performance metrics were calculated from the resulting confusion matrices.

### Predictions from individual platforms

To perform predictions on individual omics datasets, we used sparse partial least square discriminant analysis (sPLS-DA) (Lê Cao et al., 2011), as implemented in the mixOmics R package (Rohart et al., 2017). sPLS-DA is an extension of the traditional PLS approach, combining variable selection and classification in a one-step procedure. We used this method as a classification framework to predict case/control status of samples. The predictions generated from individual datasets were only used for comparison purposes with the multi-omics model.

### Prediction from integrated data

To perform predictions on the integrated datasets, we used the DIABLO framework. The implementation of the method is further detailed in (Singh et al., 2019). Briefly, DIABLO provides a classification framework based on sparse generalized canonical correlation analysis (sGCCA) (Tenenhaus et al., 2014), a multivariate dimension reduction technique that uses singular value decomposition to identify correlated variables amongst several datasets. More specifically, the method seeks linear combinations of variables (latent components) from each dataset, that are maximally correlated. This method offers the possibility to specify a design matrix, describing how the datasets should be connected (i.e.,: correlation between datasets). In this study, we used a design matrix of 0.1 to maximise the discovery of novel signatures between the datasets.

## Results

The study cohort consisted of 455 individuals (148 controls, 307 cases); detailed demographic characteristics were reported in Table 1. Gender was relatively well-balanced between the two groups, with a slightly larger proportion of females classified as cases (65.8%) compared to the control group (57.4%). As expected, the participants in the case group were significantly older (85.4 ± 4.28 years) than those classified as controls (82.8 ± 4.79 years, *p* = 2.02e^−5^), exhibited more advanced Braak stages (*p =* 4.9e^−4^) (Braak and Braak, 1991) and had a higher probability of neuritic plaques accumulation, as reflected by their higher CERAD score (*p* = 2.44e^−10^) (Fillenbaum et al., 2008). In addition, there were more carriers of at least one copy of the *APOE* ε4 allele in cases compared to the control group (*p* = 8.94e^−4^).

### The integrated dataset provided better predictions than the individual platforms

Comparing predictive capabilities (i.e.,: ability to correctly classify samples) between models built from individual datasets, we found that the SNP data provided the best *balanced accuracy* (73%), followed by the RNA data (70%). Predictions made from the methylation dataset alone, yielded a balanced accuracy of 68% and the model built with the proteomics data resulted in a 55% balanced accuracy. Overall, the integrated model provided the best predictive capabilities, showing better performance across all the metrics evaluated and resulting in a balanced accuracy of 90%, Table 2. Despite the higher prevalence of cases in the sample set (68%), the integrated model demonstrated a high sensitivity of 0.96, indicating its proficiency in correctly identifying *cases*. Specificity was measured at 0.83, supporting the model’s ability to correctly distinguish controls.

**TABLE 2:** Model performance.

Performance metric	SNP	RNA	Proteomics	CpGs	Multi-omics
Sensitivity	0.76	0.69	0.58	0.73	0.96
Specificity	0.7	0.7	0.52	0.64	0.83
Precision	0.85	0.83	0.72	0.81	0.94
Recall	0.76	0.69	0.58	0.73	0.96
F1	0.8	0.75	0.64	0.77	0.95
Accurary	0.59	0.61	0.58	0.63	0.95
Balanced Accuracy	0.73	0.7	0.55	0.68	0.9

The table above shows the performance of the single-omics models (SNP, RNA, proteomics, CpGs) and the multi-omics models. The performance metrics of each model were calculated from the corresponding confusion matrices.

### Top individual contributors of discrimination

**FIGURE 1:**
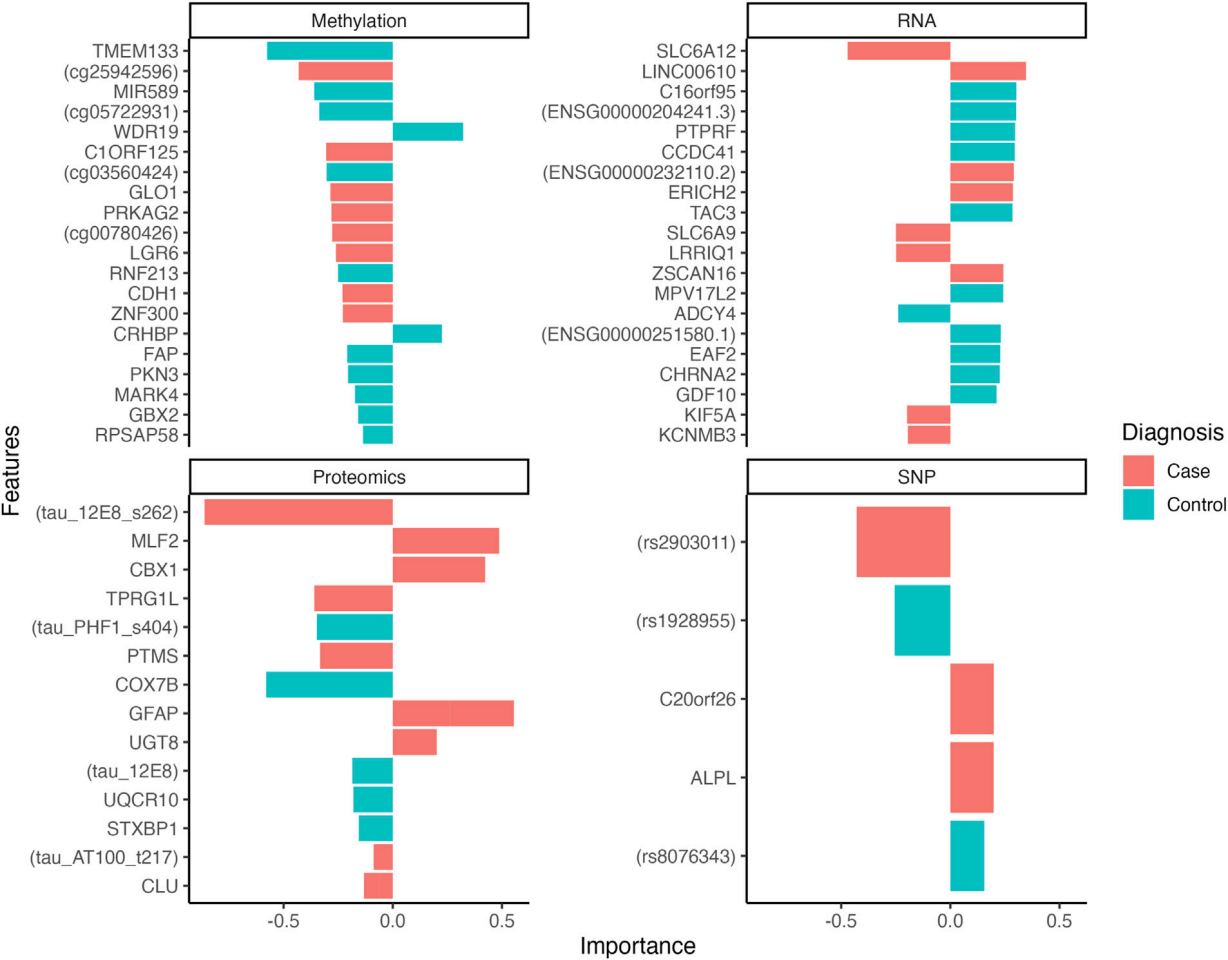
Loading plots showing the features’ weight for the first 2 latent components of multi-omics model. The weights indicate the contribution of each feature to the corresponding latent component, reflecting their importance in the discriminatory process of separating case and control samples. The color of the bars indicates whether a feature is over-represented or expressed in a specific phenotype (case or control).

The tuning phase of the multi-omics model allowed the identification of the optimal number of features to predict the case group. This corresponded to the set of features producing the best discrimination performance between cases and controls. The optimal feature panel of the integrated model consisted of 62 features, distributed as follows: 5 SNPs, 20 RNA transcripts, 20 CpGs and 17 peptides. The selected features’ contributions, as reflected by their loading weights, are shown in Figure 1 and further detailed in Supplementary Table S1. The most important features identified to separate cases and controls were the *Tau (12e8)* [*MAPT*] peptide, *ENSG00000111181* [*SLC6A12*] transcript, *cg25942596* CpG probe, the rs2903011 variant, the cg06965373 methylation probe, *Tau* [*PHF1*] peptide, *ENSG00000260456* transcript and the *rs1928955* SNP.

### Correlated features across the different datasets

**FIGURE 2:**
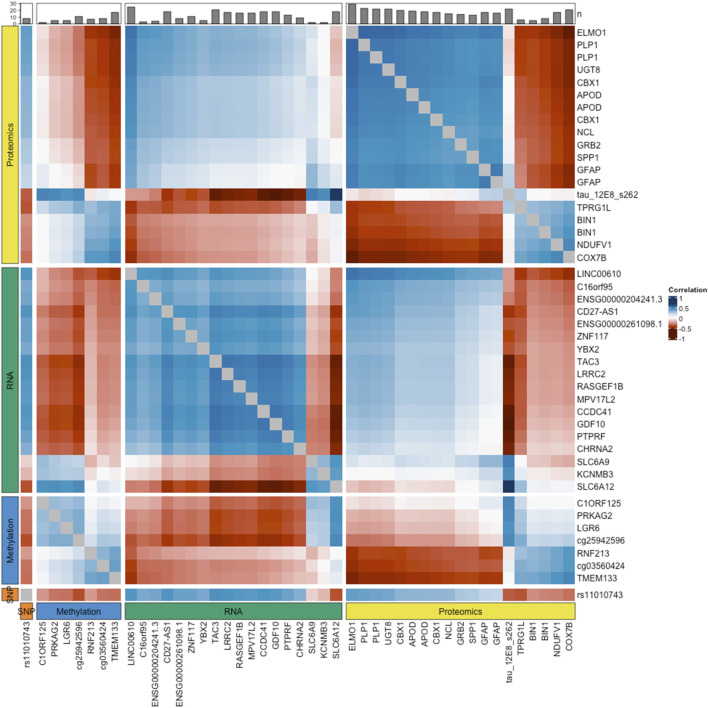
Correlation heatmap. The values shown approximate the Pearson correlation coefficients, calculated as the sum of the correlations between the original variables and each latent component in the sPLS-DA model. These values indicate how features relate to each other, reflecting their potential interactions. To facilitate the visualization of intra- and inter-omics correlations, the heatmap is divided into four panels, both vertically and horizontally, representing the four types of omics data integrated in the model. Only features with at least one correlation with an absolute coefficient above 0.5 are displayed. The bar plot at the top shows the number of these correlations for each feature, indicating their level of connectivity with other features.

Looking at cross-correlations between omics datasets, we found that the strongest correlations occurred between the *Tau (12e8)* peptide and three RNA transcripts, *ENSG00000111181* [*SLC6A12*] (r = 0.69), *ENSG00000107623* [*GDF10*] (r = −0.58) and *ENSG00000173588* [*CCDC41*] (r = −0.57). Counting the number of correlated features in each dataset, we found that the proteomics and RNA datasets were the most highly correlated datasets with 66 and 52 correlated features (absolute Pearson correlation≥0.5), respectively. At the feature level, the three most correlated variables were the *ELMO1* peptide, ENSG00000166863 [TAC3] RNA transcript and the *Tau (12e8)* peptide, with a total of 14, 10 and 9 correlations (abs(r)≥0.5), respectively. The heatmap presented in Figure 2 depicts the relationships between variables, within and across the four omics datasets.

## Discussion

Continuous technological improvements along with the development of large initiatives such as the ROS and MAP cohorts have dramatically increased the availability of multi-omics data. In this study we used a well-establish framework, DIABLO (Singh et al., 2019), to integrate multiple omics datasets and identify molecular signatures specific to AD cases and healthy control.

This framework uses a multivariate dimension reduction technique (Singular Value Decomposition) to maximise the correlated information between omics datasets. As such, it can be used to fulfill two functions: 1) as a discovery framework, to identify relevant biomarkers associated with a specific phenotype and 2) as a predictive framework. Therefore, this type of integrative approach can help achieve a more comprehensive understanding of molecular changes contributing to disease development as well as guide the development of predictive models. In the case of AD, clinical diagnosis is commonly derived from formal neuropsychiatric assessments to evaluate cognition, and a definite diagnosis can only be made post-mortem, with an autopsy revealing the presence of tau tangles and amyloid plaques. Therefore, non-invasive predictive models offer the promise of vastly improving disease detection by providing earlier intervention opportunities.

The results in this study demonstrated that we could extract multi-omics signatures to separate cases (MCI/AD) from controls (NCI). The signatures included features across the four types of omics data investigated, highlighting the tight inter-relationships and possible interactions existing between the biological layers. Amongst the major contributors in predictions, we could retrieve biomarkers known to be involved in key neurodevelopmental processes such as Tau related peptides, transcripts related to solute carrier (*SLC6A12*) and Growth and Differentiation Factor 10 (*GDF10*). The *SLC6A12* gene, for example, is a neurotransmitter transporter which has recently been screened as a hub gene, showing high expression in AD patients (Zou et al., 2023) studies have shown *GDF10* had an important role in supporting neuronal survival (Li et al., 2010) and reducing neuroinflammation (Li et al., 2015).

While supporting evidence exist for some of the main features identified, a number of key contributors identified correspond to biomarkers with unknown functions. Interestingly, most of these uncharacterised features were identified from the integrated dataset but were not detected when looking at individual omics, suggesting a synergistic role across the biological layers. Their limited effect, in isolation, could also explain the lack of annotation associated with these features.

The framework used in this study can allow for both discovery and classification/prediction; however, it is important to note that a compromise needs to be achieved between these two tasks. As further elaborated in Singh et al. (2019), the weightings defined in the design matrix plays an important role in the model’s abilities and functions. In the context of this study, we opted for a design with small weights (0.1), in order to maximise classification accuracy. This design resulted in models with highly predictive signatures but with a limited ability to extract the correlation structure from the datasets. A design matrix with larger weight values could facilitate further exploration of the interactions and relationships among the datasets, providing a more global perspective of the system and help reveal the complex mechanisms at play.

While the presented study provides valuable insights is essential to acknowledge its limitations. Each omic dataset was individually pre-processed and subject to a preliminary feature selection, in order to maintain a reasonable computational runtime for the integrated model. Although this approach effectively prevented the introduction of non-informative features in the model, it may, however, introduce biases and potentially limit the discovery of multi-omics signatures, especially those with a purely synergetic role.

Moreover, the model considered only features from the four datasets presented and did not account for the potential effects of other covariates. Incorporating additional metadata, for example, ‘age,’ which is a major risk factor for AD (Guerreiro and Bras, 2015; Hou et al., 2019), or imaging data could significantly enhance the model’s predictive power. Incorporating imaging data could be particularly beneficial, as it can provide valuable insights into structural and functional brain changes associated with AD and is a central tool for accurate diagnosis (Johnson et al., 2012; van Oostveen and de Lange, 2021). Future research could explore the incorporation of extra covariates by creating a synthetic dataset as an additional omics layer within the framework. While this endeavour was beyond the scope of the current study, it represents a promising avenue for further investigation. Finally, the relationships between the different biological layers could be further refined. The connectivity and directionality of the underlying biological networks are extremely complex and dynamic. While the use of an arbitrary design matrix to model these interactions can provide useful insights, as demonstrated in this study, novel solutions are needed to better consider the relationships between the integrated biological data.

The study demonstrates the effectiveness of integrating multiple data sources to identify robust biomarker panels and facilitate the molecular diagnostic of a complex disease such as AD. Moreover, the results presented in this study provide valuable insights on key biological pathways in AD pathogenesis, which could help identifying potential therapeutic targets. Further validations in independent cohorts are necessary to confirm the robustness and generalisability of the identified signatures. The implications of this research extend beyond AD, as the integration of multi-omics data can be applied to other complex disorders, contributing to the advancement of precision medicine and personalised approaches to disease management.

## Conclusion

The availability of high-dimensional multi-omics data has offered unprecedented resources for predictive studies. Although there are still significant contributions to be made before omics-based diagnoses becomes utilised in a clinical practice, this work demonstrates the effectiveness of integrating multiple omics for predictive purposes, compared to relying on a single source of data. The highly predictive molecular signatures identified can help improve our understanding of the key molecular mechanisms driving disease development.

## Data Availability

Publicly available datasets were analyzed in this study. This data can be found here: https://adknowledgeportal.synapse.org/, https://doi.org/10.7303/syn10468856, https://doi.org/10.7303/syn3157275, https://doi.org/10.7303/syn3157325, https://doi.org/10.7303/syn3388564.
